# Supplementary material for: Impacts of Drought Stress and Mycorrhizal Inoculation on the Performance of Two Spring Wheat Cultivars
Source: Plants (Basel). 2022 Aug 24;11(17):2187. doi: 10.3390/plants11172187 (PMC9460616; doi:10.3390/plants11172187)
Supplement: Supplementary file 1 [file plants-11-02187-s001.zip › plants-1852143-supplementary.pdf]

Supplement:

Impacts of Drought Stress and Mycorrhizal Inoculation on the Performance of Two Spring Wheat Cultivars

Caroline Pons, Caroline Müller

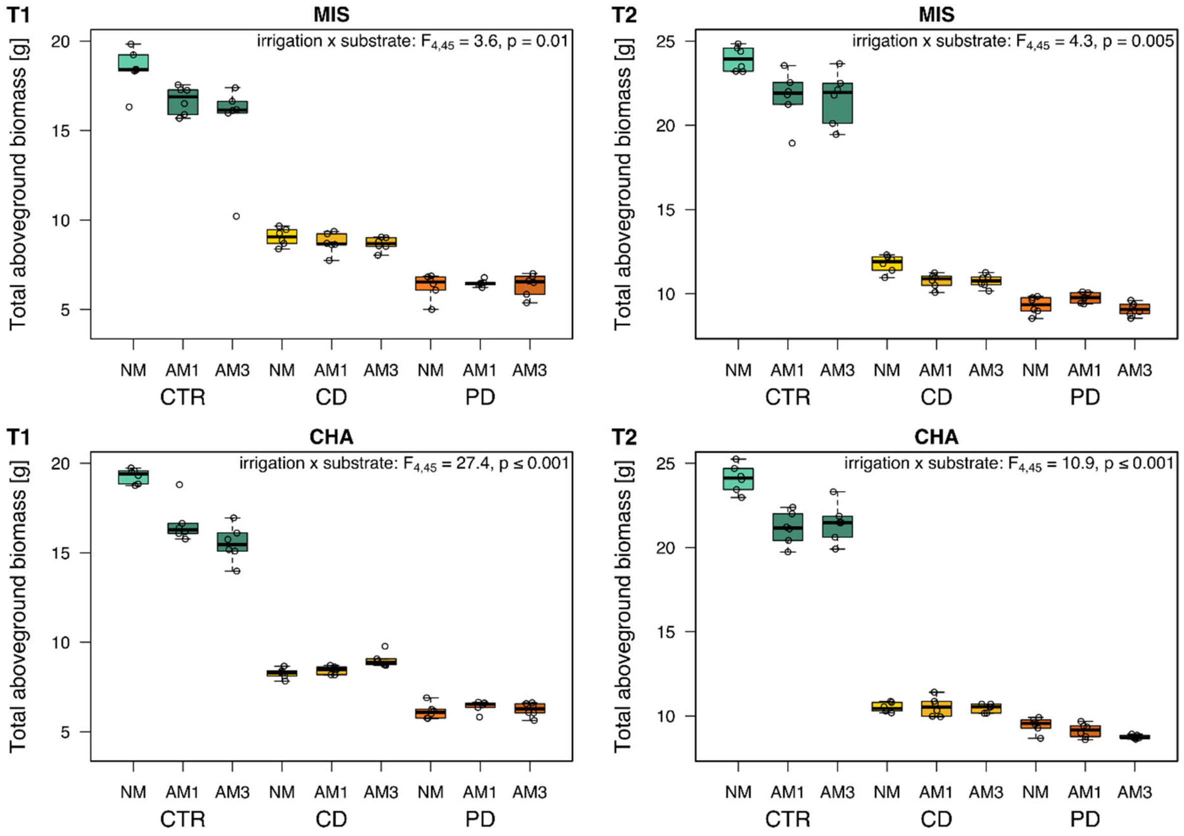

Figure S1. Total aboveground biomass from two wheat cultivars [*Triticum aestivum*; Chamsin (CHA), Mistral (MIS)] 67 days post sowing. Cultivation occurred under different irrigation conditions [control (CTR), continuous drought (CD), and pulsed drought (PD), factor: irrigation]. Plants were either non-mycorrhized (NM), mycorrhized with a single fungus (AM1; *Rhizoglomus irregulare*) or with a mixture of three fungi (AM3; *R. irregulare, Funneliformis mosseae, F. caledonium*) (factor: substrate). Data are given as box–whisker plots with interquartile ranges (IQR; boxes) including medians (horizontal lines), means (triangles), and whiskers (extending to the most extreme data points with maximum 1.5 times the IQR); individual values are given as open circles. The results of linear models are shown comprising the remaining significant terms after model simplification; n = 6.

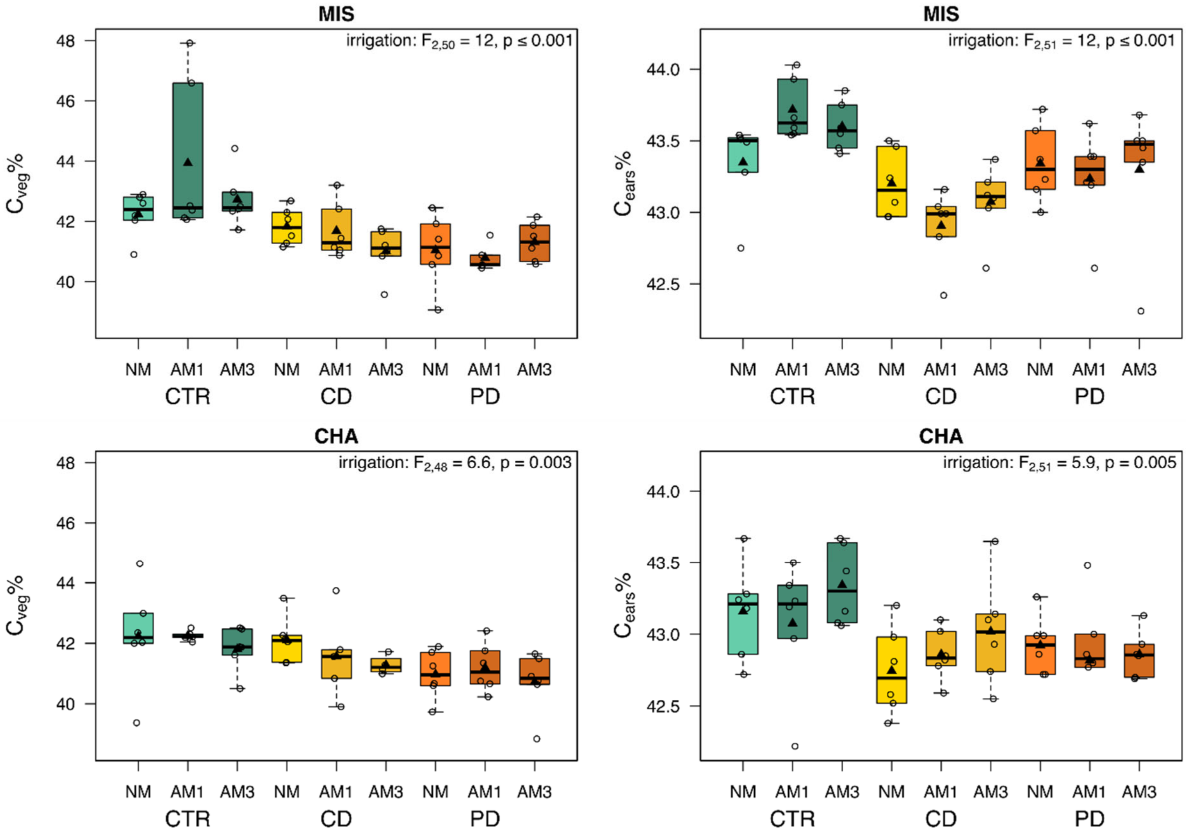

Figure S2. Total carbon (C) content of vegetative biomass (stems and leaves) and ears from two wheat cultivars [*Triticum aestivum*; Chamsin (CHA), Mistral (MIS)] 67 days post sowing. Cultivation occurred under different irrigation conditions [control (CTR), continuous drought (CD), and pulsed drought (PD), factor: irrigation]. Plants were either non-mycorrhized (NM), mycorrhized with a single fungus (AM1; *Rhizoglomus irregulare*) or with a mixture of three fungi (AM3; *R. irregulare, Funneliformis mosseae, F. caledonium*) (factor: substrate). Data are given as box–whisker plots with interquartile ranges (IQR; boxes) including medians (horizontal lines), means (triangles), and whiskers (extending to the most extreme data points with maximum 1.5 times the IQR); individual values are given as open circles. The results of linear models are shown comprising the remaining significant terms after model simplification; n = 6.

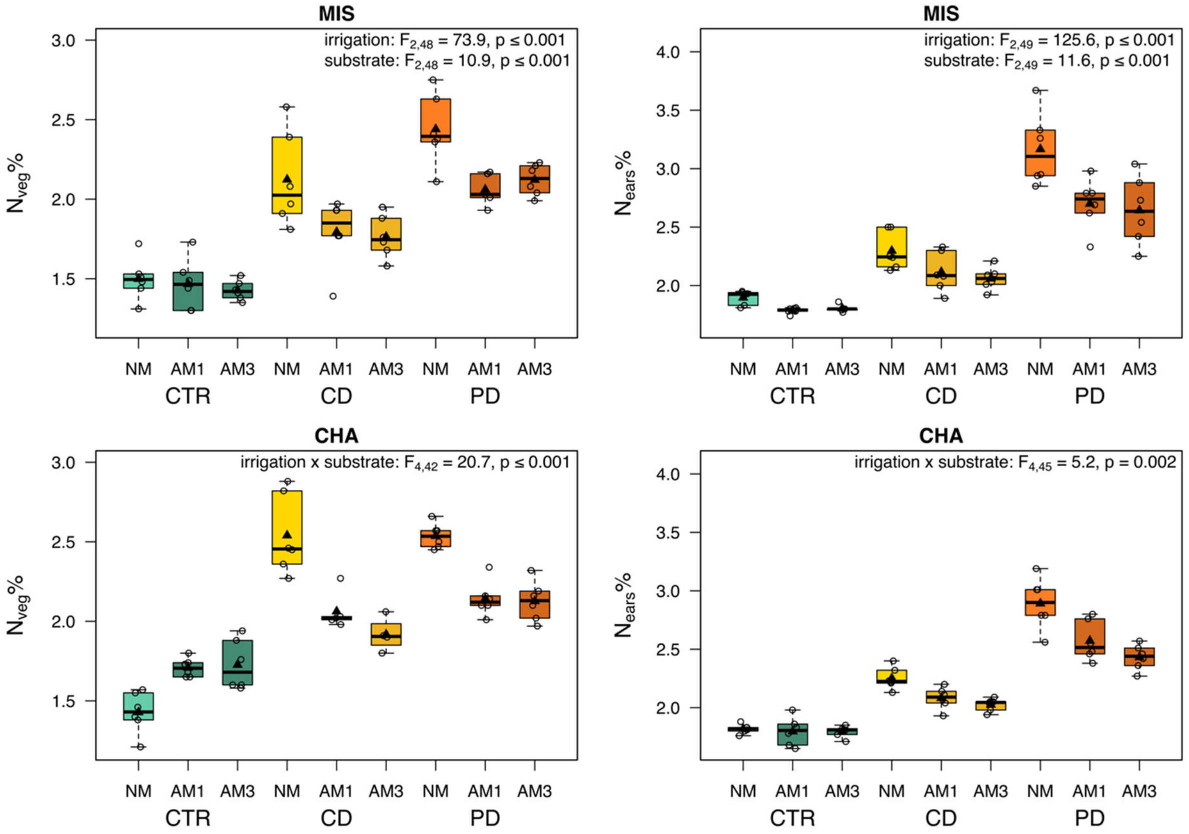

Figure S3. Total nitrogen (N) content of vegetative biomass (stems and leaves) and ears from two wheat cultivars [*Triticum aestivum*; Chamsin (CHA), Mistral (MIS)] 67 days post sowing. Cultivation occurred under different irrigation conditions [control (CTR), continuous drought (CD), and pulsed drought (PD), factor: irrigation]. Plants were either non-mycorrhized (NM), mycorrhized with a single fungus (AM1; *Rhizoglomus irregulare*) or with a mixture of three fungi (AM3; *R. irregulare, Funneliformis mosseae, F. caledonium*) (factor: substrate). Data are given as box–whisker plots with interquartile ranges (IQR; boxes) including medians (horizontal lines), means (triangles), and whiskers (extending to the most extreme data points with maximum 1.5 times the IQR); individual values are given as open circles. The results of linear models are shown comprising the remaining significant terms after model simplification; n = 4-6.

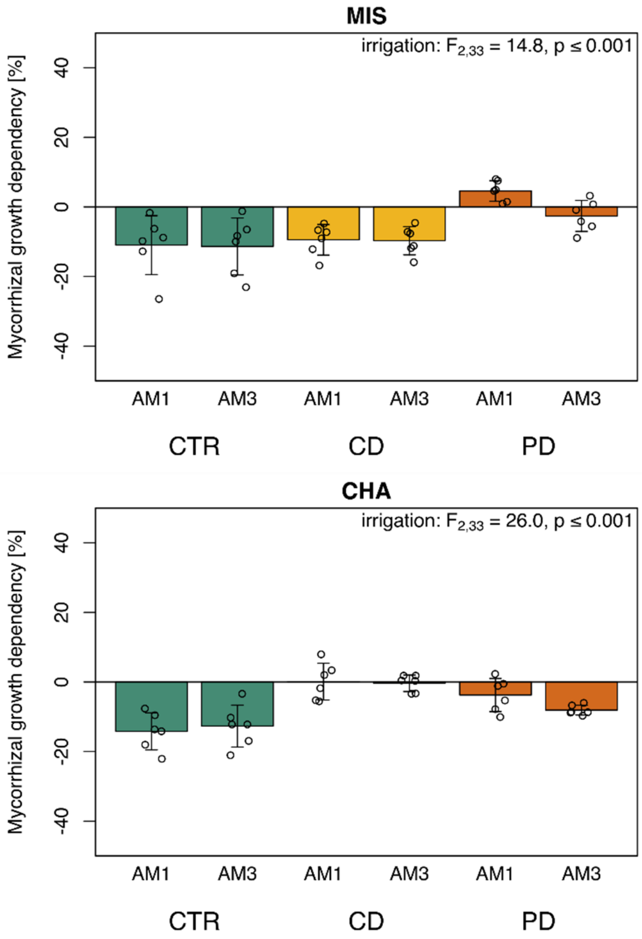

Figure S4. Mycorrhizal growth dependency [100*(aboveground dry biomass of AM plant – averaged aboveground dry biomass of NM plants) / aboveground dry biomass of NM plant)] of total aboveground biomass from two wheat cultivars [*Triticum aestivum*; Chamsin (CHA), Mistral (MIS)] 113 days post sowing. Cultivation occurred under different irrigation conditions [control (CTR), continuous drought (CD), and pulsed drought (PD), factor: irrigation]. Plants were either non-mycorrhized (NM), mycorrhized with a single fungus (AM1; *Rhizoglomus irregulare*) or with a mixture of three fungi (AM3; *R. irregulare, Funneliformis mosseae, F. caledonium*) (factor: substrate). Data are given as bar plots (mean) and standard deviation; individual values are given as open circles. The results of linear models are shown comprising the remaining significant terms after
